# Differential Effects of Inactivation of Discrete Regions of Medial Prefrontal Cortex on Memory Consolidation of Moderate and Intense Inhibitory Avoidance Training

**DOI:** 10.3389/fphar.2017.00842

**Published:** 2017-11-17

**Authors:** María E. Torres-García, Andrea C. Medina, Gina L. Quirarte, Roberto A. Prado-Alcalá

**Affiliations:** Departamento de Neurobiología Conductual y Cognitiva, Instituto de Neurobiología, Universidad Nacional Autónoma de México, Querétaro, Mexico

**Keywords:** medial prefrontal cortex, overtraining, memory consolidation, anterior cingulate cortex, prelimbic cortex, infralimbic cortex, inhibitory avoidance, state-dependent learning

## Abstract

It has been found that the medial prefrontal cortex (mPFC) is involved in memory encoding of aversive events, such as inhibitory avoidance (IA) training. Dissociable roles have been described for different mPFC subregions regarding various memory processes, wherein the anterior cingulate cortex (ACC), prelimbic cortex (PL), and infralimbic cortex (IL) are involved in acquisition, retrieval, and extinction of aversive events, respectively. On the other hand, it has been demonstrated that intense training impedes the effects on memory of treatments that typically interfere with memory consolidation. The aim of this work was to determine if there are differential effects on memory induced by reversible inactivation of neural activity of ACC, PL, or IL produced by tetrodotoxin (TTX) in rats trained in IA using moderate (1.0 mA) and intense (3.0 mA) foot-shocks. We found that inactivation of ACC has no effects on memory consolidation, regardless of intensity of training. PL inactivation impairs memory consolidation in the 1.0 mA group, while no effect on consolidation was produced in the 3.0 mA group. In the case of IL, a remarkable amnestic effect in LTM was observed in both training conditions. However, state-dependency can explain the amnestic effect of TTX found in the 3.0 mA IL group. In order to circumvent this effect, TTX was injected into IL immediately after training (thus avoiding state-dependency). The behavioral results are equivalent to those found after PL inactivation. Therefore, these findings provide evidence that PL and IL, but not ACC, mediate LTM of IA only in moderate training.

## Introduction

A large body of research has shown that interference with neural activity shortly after a learning experience results in a significant deficiency of memory consolidation (McGaugh, 1966,^ 2000^; Lechner et al., 1999; Izquierdo and McGaugh, 2000), lending strong support to the consolidation hypothesis put forward by Müller and Pilzecker (1900). This hypothesis implies that memory fixation requires time (consolidation) and that memory is vulnerable during the period of consolidation. This hypothesis, however, does not account for memory storage under some conditions of learning.

It has been found that varying the amount of training has important consequences on memory processes. When learning is brought about through intense training, memory formation is guarded against a host of amnestic treatments (For a review see Prado-Alcalá et al., 2012).^1^ This protective effect has been consistently found after training of instrumental tasks, where a reinforcer is available after performance of a specific response (Prado-Alcalá and Cobos-Zapiaín, 1977, 1979; Prado-Alcalá et al., 1980). This effect has also been described in tasks that entail both classical and instrumental components such as active and inhibitory avoidance (IA). In these cases, the animal is exposed to a conditioned stimulus and then to an unconditioned aversive stimulus, regardless of its behavior. However, after training, the animals can avoid the aversive stimulation by performing an instrumental response before the onset of the stimulus. Thus, interfering with serotonergic activity impairs both acquisition and retention of active avoidance after training with relatively low foot-shock intensities, but not when training with higher foot-shock intensities (Galindo et al., 2008). Similarly, electrolytic lesions of lateral and basal nuclei of the amygdala impaired acquisition of a Sidman avoidance task but enhanced training protected performance of this task (Lazaro-Muñoz et al., 2010).

Systemic amnestic treatments also impede memory consolidation of IA but, again, no such deficit is produced after intense training (Durán-Arévalo et al., 1990; Cruz-Morales et al., 1992; Solana-Figueroa et al., 2002; Díaz-Trujillo et al., 2009). The same is true when treatments are administered to the striatum (Giordano and Prado-Alcalá, 1986; Pérez-Ruiz and Prado-Alcalá, 1989; Salado-Castillo et al., 2011), hippocampus (Quiroz et al., 2003; Garín-Aguilar et al., 2014), amygdala (Parent et al., 1992, 1994; Thatcher and Kimble, 1966), and substantia nigra (Cobos-Zapiaín et al., 1996).

The medial prefrontal cortex (mPFC), which includes the anterior cingulate (ACC), prelimbic (PL), and infralimbic (IL) regions (Heidbreder and Groenewegen, 2003; Vertes, 2004, 2006), has received a good deal of attention in relation to its involvement in classical fear conditioning (Mello e Souza et al., 1999; Yang and Liang, 2014; Zhang et al., 2011), and it has been suggested that these regions participate differentially across the various stages of memory of fear conditioning (Giustino and Maren, 2015). Thus, ACC has been associated with acquisition (Sacchetti et al., 2003; Tang et al., 2005; Bissière et al., 2008), PL with expression (retrieval) (Blum et al., 2006; Vidal-Gonzalez et al., 2006; Corcoran and Quirk, 2007) and IL with the process of extinction (Quirk and Mueller, 2008) and control of fear (Sotres-Bayon and Quirk, 2010). However, literature on the participation of the mPFC in memory processes related to instrumental performance is scarce. It has been reported that electrolytic lesions of IL, but not of PL, produce a deficit of the instrumental component involved in retention of step-down IA (Jinks and McGregor, 1997).

To the best of our knowledge, the protective effect on learning and memory of enhanced training has not been studied in relation to selective inactivation of neural activity of ACC, PL, and IL. For this reason, and because of the differential functional attributes that have been described within the mPFC, we deemed it important to explore the effects of temporary inactivation of the three regions of mPFC on memory consolidation of moderate and intense IA training. We hypothesized that transient inactivation of AC, PL, and IL would produce differential effects on memory consolidation of IA, and that intense training would offset potential deficiencies produced by such inactivation.

## Materials and Methods

This section describes the procedures common to all the experiments of this study. Other procedures characteristic of particular experiments will be described where appropriate.

### Subjects

Male Wistar rats (300–350 g) from the breeding colony at the Instituto de Neurobiología, Universidad Nacional Autónoma de México, were individually housed with water and food *ad libitum* and maintained in a room with a 12 h/12 h light-dark cycle (lights on at 7:00 h). The temperature of the room was 23 ± 1°C. The rats were randomly assigned to each group, and training and testing were performed during the light phase of the cycle, between 8:00 am and 12:00 pm. The experimental protocol was approved by the Animal Ethics Committee of Instituto de Neurobiología, Universidad Nacional Autónoma de México and complied with the Guide for the Care and Use of Laboratory Animals (National Research Council (US) Committee for the Update of the Guide for the Care and Use of Laboratory Animals, 2011).

### Surgery

Rats were anesthetized with sodium pentobarbital (50 mg/kg, ip), injected with atropine (1 mg/kg, ip) to prevent obstruction of the respiratory tract, and their heads were positioned on a stereotaxic frame (Stoelting Co., United States). The tips of the bilateral stainless steel guide cannula (length: 8 mm for ACC and 10 mm for PL and IL; 23-gauge) were aimed 1 mm above ACC (+2.8 mm from bregma; ±0.4 mm from midline; -1.4 mm below skull), PL (+3.0 mm from bregma; ±0.7 mm from midline; -3.2 mm below skull), or IL (+3.0 mm from bregma; ±0.6 mm from midline; -4.2 mm below skull surface) (Paxinos and Watson, 2007). The cannulae were affixed to the skull using one jewelry screw and dental cement. Stylets (8 mm-long for ACC, and 10 mm-long for PL and IL) were inserted into each cannula to maintain patency and were removed, and placed back, during the manipulation sessions and for the administration of treatments. After surgery, the animals received 1.0 ml of 0.9% saline solution, ip, and were kept in an incubator until fully recovered from anesthesia. Following surgery, rats were allowed to recover for 7 days before initiation of training. During this period, each animal was handled by the experimenter, gently touching and holding the rat for approximately 5 min on three consecutive days.

### Apparatus

The rats were trained in an IA apparatus consisting of two compartments separated by a sliding door. The safe compartment (30 cm × 30 cm × 30 cm) had a lid and walls made of transparent red-colored acrylic, with a floor made of stainless steel bars (6 mm in diameter, 9 mm apart). This compartment was illuminated by a 10-W light bulb located in the center of its lid. The other, non-illuminated shock compartment (30 cm long) had front and back walls and floor made of stainless steel plates with side walls and lid constructed of transparent red-colored acrylic. The walls and floor were shaped like a trough, 20 cm wide at the top and 8 cm wide at the bottom. In the middle of the floor, a 1.5 cm slot separated the two stainless steel plates that make up the walls and floor. Upon entering the non-illuminated compartment, the rats were in contact with both plates through which a foot-shock could be delivered. A square-pulse stimulator (Grass model S-48), in series with a constant current unit (Grass model CCU-1), generated the foot-shock. Shock delivery and measurement of latencies to cross from one compartment to the other one were accomplished by use of automated equipment. Both compartments were wiped with 10% alcohol before and after each rat occupied it. The apparatus was located inside a dark, sound-proof room provided with background masking noise.

### Training and Testing of Inhibitory Avoidance

On the day of training, each rat was placed inside the safe compartment, and 10 s later the door between the two compartments was opened. The latency to cross from the safe compartment to the shock compartment is referred to as the training latency. Once the animals crossed to this compartment the door was closed and foot-shock of 1.0 or 3.0 mA was delivered (a train of 50 ms square pulses at 10 Hz). Five seconds later the door was reopened, allowing the rat to escape to the safe compartment, and then the stimulator was turned-off; this latency is referred to as the escape latency. After 30 s in the safe compartment, the rat was placed back in its home cage. Retention of the task was measured 48 h after training; in some cases retention was recorded both at 30 min (during encoding acquisition) and 48 h after training in the same animals. In these retention sessions, the same procedure as in training was followed except that the foot-shock was omitted. If the rat did not cross within 600 s, the session ended and a score of 600 was assigned.

### Treatments

Tetrodotoxin (TTX) was used to inactivate the target areas; it reversibly blocks voltage-dependent sodium channels, thus preventing the generation and propagation of action potentials (Fozzard and Lipkind, 2010). The simultaneous bilateral infusions of TTX (Sigma, C11H17N3O8, T8024; 0.3 μg/hemisphere, dissolved in 0.3 μL of isotonic saline) or an equal volume of the vehicle (VEH) into ACC, PL, or IL were made 25 min before training. In additional groups of rats, the same dose of TTX or VEH was administered into IL immediately after training. The infusion rate was 0.3 μL/min and was controlled by an automated microinfusion pump (WPI, model 220i). At the end of the infusion, the injection needles, which protruded 1.0 mm beyond the tip of the cannulae, remained inside the guide cannulae for 60 s to minimize backflow. The injection procedure was carried out in a different room from that in which training and testing took place.

### Histology

**FIGURE 1:**
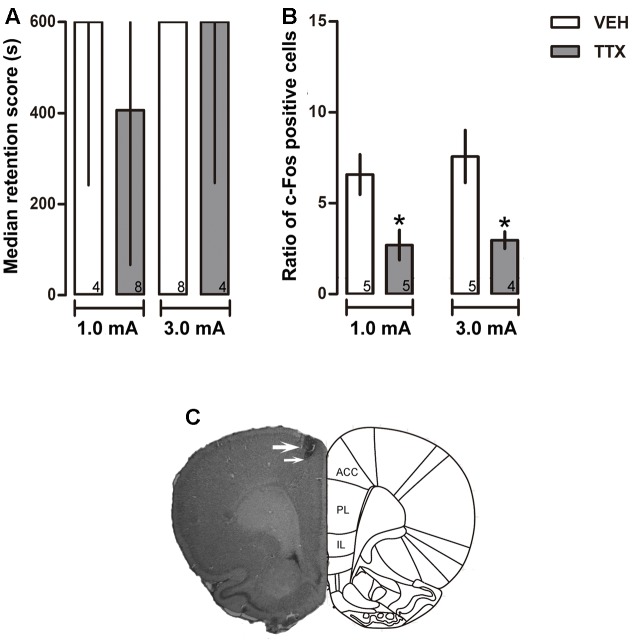
Anterior cingulate cortex. **(A)** Median retention scores (with interquartile ranges) of groups of rats trained in one-trial IA and injected 25 min before training with the vehicle solution (VEH) or TTX into anterior cingulate cortex (ACC). TTX was ineffective in altering memory consolidation of moderate (1.0 mA) or intense (3.0 mA) training. **(B)** TTX produced a reduction of c-Fos expression close to the injector tip. ^∗^*p* < 0.05 vs. VEH. **(C)** Representative photomicrograph showing location of cannula (thick arrow) and injection needle tips (thin arrow). Numbers inside bars represent sample size.

**FIGURE 2:**
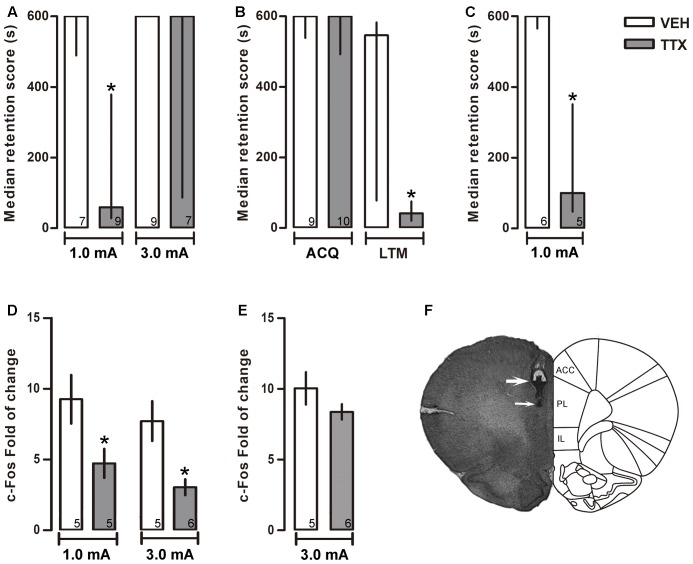
Prelimbic cortex. Median retention scores (with interquartile ranges) of groups of rats trained in one-trial IA and injected 25 min before training with VEH or TTX into prelimbic cortex (PL). **(A)** TTX impaired memory consolidation of moderate (1.0 mA), but not of intense (3.0 mA), training. **(B)** TTX did not interfere with acquisition (ACQ) but significantly impaired long-term memory (LTM). **(C)** TTX did not produce state-dependency; TTX and VEH were administered twice, 25 min before training and 25 min before the retention test. A retention deficit was observed only after the TTX treatment. **(D)** TTX produced a reduction of c-Fos expression close to the injector tip. ^∗^*p* < 0.05 relative to VEH. **(E)** TTX did not change c-Fos expression beyond 600 μm below of cannula tip. **(F)** Representative photomicrograph showing location of cannula (thick arrow) and injection needle tips (thin arrow). Numbers inside bars represent sample size.

**FIGURE 3:**
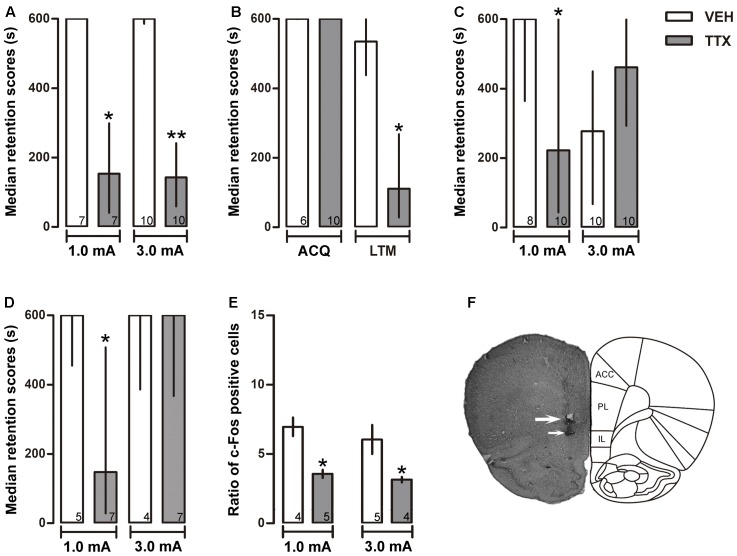
Infralimbic cortex. Median retention scores (with interquartile ranges) of groups of rats trained in one-trial IA and injected 25 min before training with VEH or TTX into infralimbic cortex (IL). **(A)** TTX impaired retention of moderate (1.0 mA) and intense (3.0 mA) training. **(B)** TTX did not interfere with acquisition but significantly impaired LTM. **(C)** TTX and VEH were administered twice, 25 min before training and 25 min before the test of retention. A retention deficit was observed in the moderate-trained TTX group, and state-dependency was produced in the intense-trained TTX group that showed good retention of the task. **(D)** Immediate post-training administration of TTX produced a deficit in memory consolidation after moderate training (1.0 mA) but not after intense training (3.0 mA). **(E)** TTX produced a reduction of c-Fos expression close to the injector tip. ^∗^*p* < 0.05 relative to VEH. **(F)** Representative photomicrograph showing location of cannula (thick arrow) and injection needle tips (thin arrow). Numbers inside bars represent sample size.

The rats were anesthetized with sodium pentobarbital (125 mg/kg) and were perfused intracardially with 0.9% saline solution followed by 4% formalin. The brains were removed and immersed in a 4% formaldehyde solution for at least 5 days. Sections were cut (50 μm thick) on a cryostat and stained with cresyl violet. The sections were examined under a light microscope, and the location of the injection needle tips was determined. The data of rats with cannula tips outside the target areas were not included in the statistical analyses. **Figures 1C**, **2F**, and **3F** show examples of cannula tip sites in ACC, PL, and IL, respectively.

### c-FOS Immunohistochemistry

To evaluate the blocking effect of TTX on neural activity in each of the three regions of mPFC that were studied, we used immunohistochemistry to detect c-Fos, as this protein is commonly used as a marker of such activity (Sagar et al., 1988; Herrera and Robertson, 1996; Willoughby et al., 1997). To this end, for each region a group of rats was trained with 1.0 or 3.0 mA, and half the group was treated with TTX or VEH, as described above, but retention of the task was not measured. A group of naïve animals (*n* = 6), used to obtain the basal number of c-Fos-positive cells, was kept under identical living conditions as those of the rest of the groups, but they never left the bioterium, except for sacrificing. One hour after training, the animals were anesthetized with sodium pentobarbital (125 mg/kg) and transcardially perfused with physiological saline followed by 4% PFA (pH 9.5, 10°C). The brains were removed and stored in the fixing solution for 4 h, then in 15% sucrose overnight followed by 30% sucrose; solutions were kept at 4°C. Three days later, four serial coronal sections (30 μm in thickness) were obtained at -20°C from ACC, PL, and IL and kept in a cryoprotectant solution (30% ethylene glycol and 20% glycerol in 0.05 M sodium phosphate buffer) at -20°C until histochemical processing. The brain slices were successively incubated in PB 0.1 M for 20 min, H_2_O_2_ 0.03% for 10 min, NaBH_4_ 1% for 6 min, and NGS 3% for 30 min. They were then incubated for 48 h at 4°C in a c-Fos polyclonal antibody (Anti-c-Fos rabbit, 1:5000, Abcam, Cambridge, MA, United States), followed by 1 h incubation in goat anti-rabbit biotinylated secondary antibody (BA-1000, 1:500; Vector Laboratories, Burlingame, CA, United States), 1 h in a Vectastain ABC Kit (Vector Laboratories, Burlingame, CA, United States), and 10 min in DAB solution (0.03% H_2_O_2_, NAS). The brain slices were placed on glass slides, dehydrated progressively with alcohol followed by the clearing agent xylene, and then covered with Entellan^®^.

Digital images were obtained with a Leica AF6000 Microsystem (Leica, Germany) using a 10× objective. c-Fos-positive cell count was automatically performed with the “cell counter” plug-in using the ImageJ software (NIH)^2^. Three counting boxes (100 μm × 100 μm) were positioned horizontally and centered 100 μm below the bilateral injection needle tracks; thus, six images per animal were analyzed. Because ACC, PL, and IL are next to each other along a dorsal-ventral dimension, it was important to assess the possibility that TTX might have had an effect due to diffusion from the target region to its neighboring ventral region. To this end, a counting box was positioned at 600 μm below each injection needle-tip track of the PL group. The expression level of c-Fos in each brain region for each group was expressed as the ratio of averaged count of c-Fos-positive cells of each rat for each group divided by the average count of c-Fos-positive cells for the corresponding naïve group.

### Statistical Analyses

Because the measurement of retention of the IA task was truncated at 600 s, non-parametric statistics were used in analyzing the behavioral results. Comparisons of training, escape, and retention latencies between TTX and VEH groups in each region of mPFC were carried out using the Mann–Whitney *U* test. Likewise, c-Fos-positive cell counts in TTX and VEH groups in each region of mPFC were compared with the Mann–Whitney *U* test.

## Results

### Anterior Cingulate Cortex

#### Training and Escape Latencies

The Mann–Whitney *U* test showed that there were no significant differences in latency scores between the TTX and VEH groups, regardless of foot-shock intensities that were used during training. Median training latencies of the VEH and TTX groups that had been trained with 1.0 mA were 15.85 and 30.35 s (*p* = 0.15), and for those trained with 3.0 mA were 19.70 and 31.05 s (*p* = 1.0), respectively (data not shown). Similarly, there were no significant differences in escape latencies between the TTX and VEH groups, regardless of the foot-shock intensities. Median escape latencies displayed by the 1.0 mA groups were 4.10 and 2.30 s (*p* = 0.57), respectively. In the VEH and TTX groups trained with 3.0 mA, escape latencies were 1.35 and 1.90 s (*p* = 0.68), respectively (data not shown).

#### Long-Term Memory

No significant differences between the VEH and TTX groups were evident in retention latencies measured 48 h after training, regardless of the intensity of foot-shock used for training (1.0 mA, *p* = 0.46 and 3.0 mA, *p* = 0.81) (**Figure 1A**).

#### c-Fos Immunohistochemistry

Tetrodotoxin administration into ACC induced a significant reduction of c-Fos expression relative to VEH in the groups that had been trained with 1.0 and 3.0 mA (*p* < 0.05 for each intensity) (**Figure 1B**). **Figure 1C** is a representative photomicrograph showing placement of a cannula tip in ACC.

### Prelimbic Cortex

#### Training and Escape Latencies

The Mann–Whitney *U* test showed that there were no significant differences in training latencies displayed by the 1.0 mA TTX (40.20 s) and VEH (26.20 s) groups (*p* = 0.18), as well as in the 3.0 mA TTX (28.80 s) and VEH (26.40 s) groups (*p* = 1.0). Similarly, there were no significant differences in escape latencies between the TTX and VEH groups, regardless of the foot-shock intensities. Median escape latencies displayed by the 1.0 mA groups were 1.80 and 1.40 s, respectively (*p* = 0.49), and median escape latencies of the TTX and VEH groups trained with 3.0 mA were 0.80 and 0.60 s, respectively (*p* = 0.27) (data not shown).

#### Long-Term Memory

The TTX group trained with 1.0 mA displayed a significantly lower score than its VEH control group (*p* < 0.05) during the 48-h retention test. In contrast, no differences were found when comparing the TTX and VEH groups trained with 3.0 mA (*p* = 0.13) (**Figure 2A**).

#### Acquisition

To evaluate whether the amnestic effect of pre-training infusion of TTX in the 1.0 mA PL group was due to interference with learning of the IA task rather than with consolidation, TTX or VEH was administered 25 min before training with 1.0 mA, and retention was measured twice: at 30 min and at 48 h after training. The results showed no significant differences between the TTX and VEH groups on the retention test run 30 min after training (*p* = 0.95) while, again, a reliable deficit was shown by the TTX group in the 48-h test (*p* < 0.05) (**Figure 2B**).

#### State Dependency

Because the TTX was administered 25 min before training, and retention was measured 48 h later in a non-drug state, it was feasible that the amnesia thus produced could have been due to a state-dependent effect. To rule out this possibility, two groups of rats were treated twice, with either TTX or VEH, 25 min before training and 25 min before retention testing. In comparison to the VEH group, the TTX group showed reliable amnesia (*p* < 0.05) (**Figure 2C**).

#### c-Fos Immunohistochemistry

Tetrodotoxin administration into PL induced a significant decrement in c-Fos expression relative to VEH in the groups that had been trained with 1.0 or 3.0 mA (*p* < 0.05, in each case) (**Figure 2D**).

As mentioned in Section “Materials and Methods,” c-Fos expression was also measured within a 100 μm × 100 μm counting box located 600 μm below the PL injector-tip tracks. We found that TTX did not interfere with c-Fos expression, as there were no significant differences between the VEH and TTX groups, *p* = 0.73 (**Figure 2E**). This finding demonstrates that the deficit in memory consolidation seen in the animals that had been trained with 1.0 mA was due to inactivation of PL and not to diffusion of the drug into the more ventrally located IL.

**Figure 2F** is a representative photomicrograph showing placement of cannula tip in PL.

### Infralimbic Cortex

#### Training and Escape Latencies

The Mann–Whitney *U* test showed that there were no significant differences in training latencies between the TTX and VEH groups, regardless of the foot-shock intensities. Median training latencies displayed by the 1.0 mA groups were 33.60 and 25.20 s (*p* = 0.80), respectively. In the VEH and TTX groups trained with 3.0 mA, training latencies were 24.20 and 23.50 s (*p* = 0.97), respectively (data not shown). Similarly, there were no significant differences in escape latencies between the TTX and VEH groups, regardless of the foot-shock intensities. Median escape latencies displayed by the 1.0 mA groups were 1.70 and 1.20 s (*p* = 0.28), respectively. In the VEH and TTX groups trained with 3.0 mA, escape latencies were 2.70 and 2.30 s (*p* = 0.65), respectively (data not shown).

As in the case of PL, TTX infusion into IL produced a significant retention deficit during the 48-h post-training session in the group that had been trained with 1.0 mA (*p* < 0.01 vs. VEH). Unexpectedly, TTX produced the same amnestic effect in the 3.0 mA group (*p* < 0.005 vs. VEH) (**Figure 3A**).

#### Acquisition

To evaluate whether the amnestic effect of pre-training infusion of TTX had been due to interference with learning rather than with consolidation, two groups of rats were trained with the low foot-shock (1.0 mA) and subjected to TTX or VEH injections into the IL 25 min before training. Retention was measured twice: at 30 min and at 48 h after training. The results showed no significant differences between the TTX and VEH groups during the retention test run 30 min after training (*p* = 0.85), while a reliable deficit was shown by the TTX group in the 48-h test (*p* < 0.02) (**Figure 3B**).

#### State Dependency

To determine whether the amnestic effect of pre-training TTX seen in the 1.0 and 3.0 mA IL groups during the 48-h retention test (**Figure 3A**) might have been due to state-dependency, two groups of rats were trained with 1.0 mA and another two groups were trained with 3.0 mA. Half of each group was treated twice with TTX and the other half with VEH, also twice, 25 min before training and 25 min before retention testing. TTX produced a significantly lower retention score relative to its VEH control group after training with 1.0 mA group (*p* < 0.05). In contrast, state-dependency was produced when 3.0 mA was used for training, as there were no reliable differences in retention scores between the TTX and VEH groups that had been trained with 3.0 mA (*p* = 0.11) (**Figure 3C**).

#### Post-training TTX Administration into IL

The results of the preceding experiment showed that pre-training TTX infusion into the IL produced a clear state-dependent effect when training was conducted with 3.0 mA, but not when 1.0 mA was used. Thus, the apparent amnestic effect produced after a single pre-training TTX infusion (**Figure 3A**) could be explained by the interaction of the high foot-shock and the differential pharmacological state of the IL cortex during training (drugged state) and retention testing (non-drugged state). This outcome did not allow us to answer the question of whether IL has a role in memory consolidation when a high aversive stimulation is used to produce learning. To shed light into this matter, we decided to study the effects of IL inactivation with TTX induced after training, thus avoiding the confounding effect of state-dependency. To this end, two groups of rats were trained with 1.0 mA and another two groups were trained with 3.0 mA. Half of each group was treated with TTX and the other half with VEH. The infusions were made immediately after training. A significant retention deficit was observed in the TTX group that had been trained with 1.0 mA (*p* < 0.03 vs. VEH), whereas no significant differences between the TTX and VEH groups trained with 3.0 mA were found (*p* = 0.11) (**Figure 3D**).

#### c-FOS Immunohistochemistry

The infusion of TTX into the IL induced a significant decrement in c-Fos expression relative to VEH in the groups that had been trained with 1.0 or 3.0 mA (*p* < 0.05 for each comparison) (**Figure 3E**).

## Discussion

The main findings of this study, where TTX was administered before training, were that regardless of the intensity of training, transient inactivation of ACC did not disrupt memory consolidation of the IA task. In contrast, in PL and IL TTX produced a highly significant deficit of consolidation when the moderate foot-shock was used in training. Interestingly, the retention deficit was still evident after training with the high foot-shock when IL had been inactivated, due to state-dependency, but retention was not diminished in the PL group (**Figures 1A**, **2A**, **3A**). When TTX was administered immediately post-training into IL, it interfered with consolidation only when the moderate foot-shock was used. We suggest that these differential effects are dependent on the dissimilar connectivity of the three regions that were studied. They receive strong connections from the same thalamic regions; PL and IL receive afferents from the basolateral and basomedial nuclei of the amygdala; and PL is more densely connected to limbic cortical areas than ACC and IL (Hoover and Vertes, 2007). Further research is needed to study the contribution of these different anatomical interactions in memory consolidation of IA.

The histochemical results showed that administration of TTX in each of those cortical regions produced reliable neuronal inactivation, as evidenced by the diminished detection of c-Fos near the injector tips. Because there were no significant differences in training and escape latencies between the TTX- and VEH-treated animals, irrespective of the microinjected region or the intensity of the foot-shock used for training, the impaired retention that was found in the PL and IL groups cannot be explained by any potential deficiency of the motor or perceptual activities necessary to perform the IA task. In other words, the treated animals could cross from the safe compartment to the shock compartment, and escape from the foot-shock just as efficiently as the VEH-treated animals. What follows is a discussion focusing on relevant studies on IA.

### Anterior Cingulate Cortex

Inactivation of ACC did not interfere with memory consolidation of IA, as indicated by the high retention scores of animals trained with 1.0 and 3.0 mA. This agrees with the report of Mello e Souza et al. (1999) that intra-ACC administration of muscimol and AP5 did not impede the formation of long-term memory of this task. Our result is also congruent with the lack of impairment of memory consolidation of IA found after pre-training radiofrequency lesion (Chai et al., 2010) of ACC. Taken together, these data suggest that this region is not involved in neural activity encoding needed for memory consolidation of the CS-UCS association during training of the IA task. This interpretation must be taken cautiously, because other lines of research suggest that ACC is involved in memory consolidation of IA. Thus, infusion of the cholinergic agonist oxotremorine into ACC immediately after IA training improved memory (Malin and McGaugh, 2006) and, consistent with this finding, it was shown that pre-training and post-training infusion of scopolamine, a cholinergic antagonist, impaired memory consolidation of this task (Riekkinen et al., 1995). Furthermore, administration of a protein synthesis inhibitor into ACC or mPFC (which included PL and IL) produced a significant retention deficit of IA (Zhang et al., 2011). New studies are needed to comprehend these dissimilar results.

### Prelimbic Cortex

The findings that inactivation of PL produced a marked deficiency of retention when the low intensity foot-shock was used for training, and that it did not impede performance when the high foot-shock was used (**Figure 2A**) fit well with previous results where interference with neural activity of striatum (Giordano and Prado-Alcalá, 1986; Pérez-Ruiz and Prado-Alcalá, 1989), hippocampus (Quiroz et al., 2003; Garín-Aguilar et al., 2014), amygdala (Parent et al., 1992, 1994), and substantia nigra (Cobos-Zapiaín et al., 1996; Salado-Castillo et al., 2011) disrupted memory consolidation when IA training took place with a low intensity of aversive stimulation, but not when stimulation of relatively high intensity was used.

That the impairment in retention shown by the TTX group that had been trained with the low foot-shock was due to interference with memory consolidation, and not to a deficiency in learning, was demonstrated by the optimal performance shown by the group of animals that was tested 30 min after the administration of the drug. A deficit in consolidation became evident when this group was given a second retention test 48 h later (**Figure 2B**).

Because training took place under the influence of TTX, and retention of the task was measured when the animals were in a non-drugged condition, the possibility existed that the retention deficit observed in the low foot-shock group was due to a phenomenon of state-dependency and not to disturbance of memory consolidation. This possibility was discarded because a group of rats that was trained and tested under the same pharmacological condition exhibited a deficient retention (**Figure 2C**).

The retention deficit observed in the present study gives support to the findings of Santos-Anderson and Routtenberg (1976) and of Jinks and McGregor (1997). The former authors showed that low-level electrical stimulation of the ventral aspect of the mPFC interfered with memory consolidation of IA, and the latter found that electrolytic lesions of PL produced a deficit in IA. Together, these findings indicate that PL has similar functions to those of the striatum, hippocampus, amygdala, and substantia nigra regarding memory consolidation of IA, i.e., these structures are necessary for memory consolidation under conditions of moderate training because interference with neural activity of any one of them impedes the formation of long-term memory. On the other hand, consolidation takes place after intense training despite this interference. It has been hypothesized that these structures are not critical for mediating associative processes derived from intense training, which produces plastic changes allowing for the recruitment of other structures.

### Infralimbic Cortex

The study of IL yielded a complex set of data. In agreement with previous results, where lesions of IL produced a significant retention deficit of IA (Jinks and McGregor, 1997), we found that infusion of TTX into IL had the same detrimental effect when the low foot-shock was used for training. Contrary to our expectations, intense training did not protect memory consolidation against the inactivation produced by the TTX (**Figure 3A**). The memory test that was made 30 min post-training to the group of animals that were treated with TTX into IL yielded top retention scores, while a reliable deficit was observed 48 h later in this same group. As in the case of PL inactivation, these results indicate that learning took place and that the impaired retention was due to a failure in memory consolidation (**Figure 3B**).

When TTX was infused into IL twice (both before training and before the 48-h retention test) a retention deficit was produced in the low foot-shock group but, unexpectedly, not in the high foot-shock group (**Figure 3C**). To the best of our knowledge, this is the first time that a state-dependent effect has been found in mPFC. Thus, the question of whether IL is involved in memory consolidation of intense training could not be answered with this experimental design. This problem was solved by administering the treatments immediately after training with the low and the high intensity of foot-shock, thus avoiding the induction of state-dependency. This manipulation confirmed that inactivation of IL impedes memory consolidation when IA training is carried out with an aversive stimulus of low intensity, and it revealed that, indeed, intense training protects against the amnestic effect of inactivation produced by the TTX (**Figure 3D**).

## Conclusion

The data obtained in this experimental series indicate that (a) memory consolidation of IA is not dependent on neural activity of the ACC; (b) normal activity of PL is essential for memory consolidation of moderate IA training, but not for acquisition or for consolidation of intense training; (c) normal activity of IL is also essential for memory consolidation of moderate IA training but not for acquisition or for consolidation of intense training. Moreover, the combined effect of TTX and intense training induces state-dependency.

## Author Contributions

Designed the experiments: MT-G, AM, and RP-A. Performed the behavioral and histological experiments: MT-G and AM. Analyzed the data: MT-G and AM. Wrote and provided comments and discussion for the manuscript: MT-G, AM, GQ, and RP-A.

## Conflict of Interest Statement

The authors declare that the research was conducted in the absence of any commercial or financial relationships that could be construed as a potential conflict of interest.

## Footnotes

1 Intense or enhanced training refers to conditions where a high number of trials or training sessions are given, and, in the case of aversive conditioning, to training motivated by relatively high intensities of foot-shock. In both instances, intense training yields stronger resistance to extinction than moderate and low levels of training.

2 http://rsb.info.nih.gov/ij/
